# Analysis of microRNAs, phased small interfering RNAs and their potential targets in *Rosarugosa* Thunb.

**DOI:** 10.1186/s12864-018-5325-2

**Published:** 2019-04-18

**Authors:** Junqiang Guo, Qingyi Wang, Li Liu, Shuchao Ren, Shipeng Li, Peiran Liao, Zhigang Zhao, Chenyu Lu, Bingbing Jiang, Ramanjulu Sunkar, Yun Zheng

**Affiliations:** 1 Faculty of Information Engineering and Automation, Kunming University of Science and Technology, Kunming, 650500 China; 2 Faculty of Life Science and Technology, Kunming University of Science and Technology, Kunming, 650500 China; 3 Yunnan Key Laboratory of Primate Biomedical Research, Institute of Primate Translational Medicine, Kunming University of Science and Technology, Kunming, 650500 China; 4 Department of Biochemistry and Molecular Biology, Oklahoma State University, Stillwater, 74078 Oklahoma USA

**Keywords:** *Rosa rugosa* Thunb., High-throughput sequencing, microRNA, Phased small interfering RNA (phasiRNA), SeqTar, Degradome, Bioinformatics

## Abstract

**Background:**

MicroRNAs (miRNAs) are small non-coding RNAs that play important roles by regulating other genes. *Rosa rugosa* Thunb. is an important ornamental and edible plant, yet there are only a few studies on the miRNAs and their functions in *R. rugosa*.

**Results:**

We sequenced 10 samll RNA profiles from the roots, petals, pollens, stamens, and leaves and 4 RNA-seq profiles in leaves and petals to analysis miRNA, phasiRNAs and mRNAs in *R. rugosa*. In addition, we acquired a degradome sequencing profile from leaf of *R. rugosa* to identify miRNA and phasiRNA targets using the SeqTar algorithm. We have identified 321 conserved miRNA homologs including primary transcripts for 25 conserved miRNAs, and 22 novel miRNAs. We identified 592 putative targets of the conserved miRNAs or tasiRNAs that showed significant accumulations of degradome reads. We found differential expression patterns of conserved miRNAs in five different tissues of *R. rugosa*. We identified three hundred and thirty nine 21 nucleotide (nt) PHAS loci, and forty nine 24 nt PHAS loci, respectively. Our results suggest that miR482 triggers generations of phasiRNAs by targeting nucleotide-binding, leucine-rich repeat (NB-LRR) disease resistance genes in *R. rugosa*. Our results also suggest that the deregulated genes in leaves and petals are significantly enriched in GO terms and KEGG pathways related to metabolic processes and photosynthesis.

**Conclusions:**

These results significantly enhanced our knowledge of the miRNAs and phasiRNAs, as well as their potential functions, in *R. rugosa*.

**Electronic supplementary material:**

The online version of this article (10.1186/s12864-018-5325-2) contains supplementary material, which is available to authorized users.

## Background

Small non-coding RNAs (ncRNAs), ranging in sizes between 20 and 30 nucleotides, play crucial roles in the biological processes of almost all living organisms. MicroRNAs (miRNAs) and endogenous small interfering RNAs (siRNAs) are two types of small ncRNAs [1]. Under the action of RNA polymerase II, the MIR gene is transcribed into a primary transcript called pri-miRNA [2]. The plant pri-miRNA of typical hairpin-like structure is cleaved into a precursor (per-miRNA) of the stem-loop structure in the nucleus by Dicer Like protein (DCL1). Mature plant miRNA:miRNA* duplex excised form pre-miRNA by DCL1 and then exported to the cytoplasm [1, 3, 4]. The mature miRNA strand of duplex strand is loaded into an RNA-induced silencing complex (RISC) that normally contains an Argonaute (AGO) protein in the cytoplasm, then leads the RISC to cause site-specific repression or cleavage of the mRNA targets [5, 6]. The other strand of the duplex, called miRNA*, is degraded [7]. Most plant miRNAs recognize their target mRNAs through homology-dependent mechanism and negatively regulate their expression by guiding cleavage or translation inhibition [4, 8, 9], furthermore some miRNAs may activate their targets, especially metazoan miRNAs [10–12].

Samiliar to miRNAs, siRNAs also have significant functions in plants. PhasiRNA is a class of secondary siRNAs that are generated precisely in 21 or 24 nt phased pattern initiated at a specific position due to miRNA guided activity. A type of phasiRNAs are *trans*-acting siRNAs (tasiRNAs) because they repress their target transcripts from other loci of the genome at post-transcriptional level [13]. The primary transcripts of tasiRNAs are non-coding and used to generate double strand RNAs (dsRNAs) by RDR6 (RNA-dependent RNA polymerase 6) [14]. The dsRNAs are then cleaved by DCL4 to form phased 21 nt segments [15–17] or by DCL5 to form 24 nt phased segments as shown in rice [18, 19]. The precise phasing of tasiRNAs is guided by miRNAs [15] through either two [20] or one [18, 21–24] miRNA binding site on the primary tasiRNA transcripts. Four families of tasiRNA loci, named TAS1 to TAS4, have been identified in *Arabidopsis thaliana* [15, 17, 25]. Recent studies suggest that some coding genes, especially PPR [17, 24, 26], NB-LRR disease resistance proteins [24, 27–31], MYB transcription factors [24, 32, 33], also generate phasiRNAs, and their corresponding loci are called as PHAS loci [27]. These PHAS loci are also triggered by one or two miRNA binding sites [14, 27]. *TAS3* derived tasiARFs are the only phasiRNAs that have been validated to target ARF genes in *trans* [15, 20, 24]. The functions of most phasiRNAs are still largely unknown [14].

Rose is a generic name for a variety of rosa. *Rosa rugosa* Thunb. (*R. rugosa*) (old rose), originated in China, belongs to the Rosa family. It is a cash crop important for its fragrant and beautiful flowers [34]. In the past century, *R. rugosa* has emerged as one of the most important crops in the floriculture industry worldwide and cut *R. rugosa* accounts for nearly one third of cut flower trade in Europe [35]. In addition, *R. rugosa* possesses some unique morphological and physiological features, that include highly divergent flower colors, shapes and volatiles and recurrent flowering, which are unable to be studied in other model plant systems, like tobacco and *Arabidopsis thaliana* [36]. *R. rugosa* flowers are mainly used in food and refined *R. rugosa* otto. *R. rugosa* otto is widely used in cosmetics, foodstuff, fine chemicals and other industries. Although *R. rugosa* is very important, there are only a few studies that reported sRNAs in *R. rugosa* [34, 37].

Flowers are the most important organs in *R. rugosa* for the economic and ornamental values. Therefore, we preferentially chose to study the miRNAs and phasiRNAs in *R. rugosa* flowers. We sequenced 10 small RNA libraries from 5 tissues, i.e., roots, petals, pollens, stamens and leaves and 4 RNA-seq libraries from leaves and petals of *R. rugosa*. We found that 2000 genes have significantly different expression levels in leaves and petals of *R. rugosa*. These genes are mainly involved in different metabolic processes and photosynthesis related pathways. Comprehensive analyses of these small RNA profiles lead to identification of 25 pre-miRNAs of which 24 have not been reported, 22 novel miRNAs, 339 PHAS loci encoding 21 nt phasiRNAs, and 49 PHAS loci encoding 24 nt phasiRNAs. A degradome profile of *R. rugosa* leaf was generated for identifying targets of miRNAs and phasiRNAs. Totally, more than 19,000 putative targets for conserved miRNAs and TAS3-derived tasiRNAs were identified in the analysis. At least 592 targets have shown significant accumulation of degradome reads corresponding to the identified miRNA complementary sites. These results clearly deepen our understanding about small RNA guided gene regulations in *R. rugosa*.

## Methods

### Materials and small RNA sequencing profiles

The *R. rugosa* plants were grown in Kunming University of Science and Technology, Yunnan, China. We collected ten samples from the roots, leaves, pollen, petals, and stamens of *R. rugosa* without treatment and frozen with liquid nitrogen immediately (Additional file 1: Table S1). Before RNAs were extracted, these 10 samples were stored at -80 °C. According to the manufacturer’s protocol, we used the Trizol reagent to extracte total RNAs from samples. Based on the ratio of the optical density at 260 nm to that at 280 nm (OD260/280), the integrities of RNAs were checked by using an ultraviolet spectrophotometer (Hoefer, MA, USA). And then in view of visual comparison of the 18S and 28S ribosomal RNAs, the integrities of RNAs were also assessed by electrophoresis in a denaturing formaldehyde agarose gel. The total quantities of RNA samples with OD260/280 between 1.8 and 2.0 were examined. Samples with at least 20 µg were selected for preparation of sRNA sequencing libraries. 20 µg total RNAs dissolved in 35 µl were delivered to the sequencing facility. For small RNA-seq library generation, 1 µg of total RNA was used to prepare a small RNA library according to the TruSeq Small RNA Sample Prep Kit protocol (Illumina, San Diego, USA). Briefly, specific 5’ and 3’ Illumina RNA adapters were sequentially added to small RNA molecules. After reverse transcribed and PCR amplified, adapter-ligated molecules with 15 to 30 nucleotides long were purified by using gel electrophoresis. The small libraries were then multiplexed and sequenced by using the Illumina HiSeq 2000 sequencer. The qualities of the obtained sRNA profiles were evaluated with the FASTQC program (https://www.bioinformatics.babraham.ac.uk/projects/fastqc/). The obtained small RNA profiles had been deposited to NCBI GEO database under the accession number, GSE110490.

### RNA-seq profiles of leaves and petals in *R*.*rugosa*

Two petal and leaf samples of *R. rugosa* plants grown in Kunming University of Science and Technology, Yunnan, China were collected and put into liquid nitrogen immediately. Total RNAs of these 4 samples were extracted using the same protocol as above. The integrities of the RNAs were also checked using same protocols as above. The total RNAs of the 4 samples were sequenced using Illumina HiSeq 4000 sequencer with a 2×150 pair-end RNA-seq strategy. The obtained RNA-seq profiles were deposited into the NCBI GEO database under the accession number GSE113148.

These 4 RNA-seq profiles were assembled using Trinity (v2.0.6) [38] with the options of “–seqType fq –max_memory 400G –min_contig_length 150 –CPU 30 –min_kmer_cov 3 –min_glue 3 –bfly_opts ’-V 5 –edge-thr=0.1 –stderr”’. This results in a transcriptome with more than 370,000 transcripts. These transcripts were then aligned to NCBI nt/nr database with the options of “-e 1e-5 -m 8” to obtain their putative annotations.

The expression levels of the assembled transcripts were estimated by the align_and_estimate_abundance.pl program in the Trinity package using the options of “–seqType fq –est_method RSEM –aln_method bowtie –trinity_mode –prep_reference”.

The expression levels of transcripts in leaf and petal samples were compared with edgeR [39]. The expression levels of transcripts were filtered to keep 37,278 transcripts with average expression levels of at least 10 FPKM and standard deviations of expression levels of at least 10 FPKM. Then, the expression levels of these 37,278 were used to perform a bi-clustering analysis using the pheatmap library in R. Transcripts with FDR values smaller than 0.05 were called as deregulated. The deregulated transcripts were used to analyzed enriched GO terms and KEGG pathways using KOBAS (version 3) [40].

### Identification of conserved and novel miRNAs

By using the computational methods reported previously [41], we analyzed the small RNA libraries. Firstly, we filtered out raw reads containing more than five low scored nucleotides (< 25). After removing 3’ adaptor sequences of the remaining reads and redundant sequences, we obtained unique small RNAs. Then we removed unique sequences which could be mapped to known repeats and non-coding RNAs (snRNAs, tRNAs, rRNAs, snoRNAs) by aligning them to databases, Rfam (r11) [42], NONCODE (v3.0) [43], Silva [44], The TIGR Plant Repeat Databases [45] and Repbase (r20) [46] using SOAP2 [47]. To calculate the frequencies of conserved miRNAs, we mapped remaining reads to the miRBase database (r21) [48]. We also counted and summarized the numbers of reads that were mapped to different types of molecules (Additional file 1: Table S2).

The remaining unique sequences that could be mapped to the transcriptome, but not to pre-miRNAs, ncRNAs and repeats were aligned to the self-assembled *R. rugosa* transcriptome by SOAP2. The flanking sequences (down- and up-stream) of the matched loci were used to predict secondary structures using RNAfold [49]. We selected the structures that satisfy the following criteria as putative precursors: at least 18 paired nucleotides, only one central loops in the hairpin structure and a folding energy of smaller than -30 Kcal/mol. The small RNA sequencing reads in the 10 sRNA-seq libraries were mapped to the obtained putative precursors. Finally, novel miRNA identification and annotation were strictly based on appearance of miRNA* and predictable fold back structures for the miRNA precursor sequences, as suggested by Meyers et al. [50].

In order to identify conversed miRNAs homologs in *R. rugosa*, we downloaded all mature miRNA sequences of plant species from the miRBase (v21) [48], and removed redundant sequences as described previously [13, 41]. Then these unique miRNA sequences were mapped to the *R. rugosa* cDNAs using BLASTN. Hits with no more than two mismatches were identified. The flanking regions (80nt, 130nt, 180nt down stream and upstream) of the mapped mature miRNAs were isolated and used to predict secondary structures using the RNAfold. The predicted fold-back structures were examined for the presence of miRNA on the same arm of the hairpin as the known family members from other plants. We use MIRcheck to further evaluate these precursor sequences. The precursors were selected which have ≤ 2 bulged, ≤ 5 mismatches, or asymmetrically unpaired nucleotides, and ≤ 3 continuous mismatches within the mature miRNA.

Then the identified conserved miRNAs was compared with those reported previously [34, 37]. If the sequence of a mature miRNA was identical to the previously reported sequence, the conserved miRNA was named the same as in the previous report. The remaining conserved miRNAs were named by using upper case MIR followed by the family name, and alphabetical letters in lower case if these have not been reported earlier.

### Analysis of miRNA expression patterns in different tissues

To analyze the expression patterns of miRNAs in different tissues, we used edgeR [39] to compare the RPTM (Reads Per Ten Million sequencing tags) of mature miRNAs from different tissues. miRNAs with FDR (False Discovery Rate) less than 0.05 were considered as significantly deregulated miRNAs in different tissues.

miRNA with abundances of at least 5 RPTM in at least two of the 10 samples and standard deviation of at least 1 were selected to perform hierarchical clustering. The normalized RPTM values plus one were log scaled and applied to the pheatmap function in the pheatmap library in R to perform bi-clustering. miRNAs with RPTM mean of at least 5 in 10 samples were used to perform Principle Component Analysis (PCA). The RPTM values plus one for these miRNAs were log-scaled and used to perform PCA by the procmp function in R.

### Identification of TAS3 loci in *R*.*rugosa*

As reported previously [51, 52], tasiRNAs that were produced from TAS3 genes in *Arabidopsis* and rice were collected. These tasiRNAs were aligned to the self-assembled *R. rugosa* cDNA database with up to two mismatches allowed. Both the upstream and downstream 250 bp sequences of matched loci were cut out form cDNA.

### Identification of PHAS loci in *R*.*rugosa*

We used 10 small RNA profiles generated in this study to predict PHAS loci and phasiRNAs. The method for predicting PHAS loci and phasiRNAs were described previously [13, 41, 53]. The unique sequences in the small RNA libraries were aligned to The TIGR Plant Repeat Databases [45] and Repbase (r20) [46] to remove sRNAs mapped to repeats, then the remaining sRNAs were mapped to the cDNA of *R. rugosa* using SOAP2 [47]. In order to scan the cDNA sequences using a window of 210 nt or 240 nt (ten 21 nt or 24 nt, respectively) respectively, a self-written program was used. Because the existence of two-nucleotide over-hang at the 3’-end of siRNA duplex, a two-nucleotide positive offset was used to calculate the positions of siRNAs on the anti-sense strand [17, 24, 26, 27]. Then a *P*-value was calculated for each of the windows using a Hypergeometric test [26]. And a phase score was calculated for each position of cDNA sequences using the method in [54].

The window with a *P*-value less than 0.05 was extended 100 bp at both 5’- and 3’-ends, then the overlapped windows were merged. The *P*-values of the combined windows were used to calculate the false positive rates using the method in [55]. The merged windows with a maximal phase scores of more than 5 and FDR values less than 0.05 were reported as PHAS loci. The predicted PHAS loci were named with the contig names of self-assembled cDNAs and a unique serial number for each contig. “P21” and “P24” were added at the beginning of the predicted PHAS loci encoding 21 and 24 nt phasiRNAs. The neighboring PHAS loci were predicted as PHAS clusters if the distances between individual PHAS loci were smaller than 2000 base pairs. The phased siRNAs of the predicted PHAS loci were reported as phasiRNAs. The phasiRNAs of a PHAS loci were named by adding siR and a unique serial number to the name of the PHAS loci [13, 41, 53].

### Degradome sequencing

A leaf sample was frozen in liquid nitrogen immediately after harvesting form a *R. rugosa* plant grown in Kunming University of Science and Technology, Kunming, Yunnan, China. According to the manufacturer’s protocol, we used the Trizol reagent to extracte total RNAs from samples. The integrity of the RNA was checked with an ultraviolet spectrophotometry and Agilent 2100 BioAnalyzer. For constructing degradome library, 20 *μ*g of total RNA was annealed with biotinylated random primers. These biotin-tagged RNA fragments were then captured on streptavidin. Then the RNAs which contain 5’ -monophosphates were ligated with a 5’ adaptor. After reverse transcription and PCR amplification, libraries were sequenced using the 5’ adapter on an Illumina HiSeq 2000 sequencer with a single-end mode of 47 base pairs. The sequenced reads represented the 5’ ends of the sliced RNA fragments that were ligated with the adapters. The obtained degradome profile had been deposited to the NCBI GEO database under the accession number, GSE110494.

The quality of the obtained degradome profile was evaluated with the FASTQC program. The obtained degradome profile was processed with a self-developed pipeline. Firstly, low quality reads that have low scored nucleotides (< 30) in the first 25 nucleotides were removed from the raw degradome sequencing profile. Then the first twenty nucleotides were cut out from the remaining reads for later analysis. Next, we removed redundant sequences and calculated the frequencies of the unique sequences. Similar to sRNA profiles, unique sequences were aligned to cDNA of *R. rugosa*, Rfam (r11) [42], NONCODE (v3.0) [43], Silva [44], The TIGR Plant Repeat Databases [45] and Repbase (r20) [46] using SOAP2 [47] to calculate the numbers of reads and unique sequences mapped to different categories of molecules as well as repeats. The SOAP2 alignment result of mapping degradome sequences to *R. rugosa* cDNA was used to identify targets of miRNAs and siRNAs.

### Identification of miRNA/siRNA targets in *R*.*rugosa*

To predict the targets of miRNAs and tasiRNAs, we used the SeqTar pipeline [56]. For conserved miRNAs and tasiRNAs, targets with less than four mismatched nucleotides to miRNAs/tasiRNAs were used for later analysis. For novel miRNAs, targets with at least one valid read and no more than four mismatches were selected for subsequent analysis.

Similarly, the SeqTar algorithm was used to predict targets of phasiRNAs. Only targets that satisfy the following two conditions were used for later analysis: targets that have no mismatches or at least 1 valid degradome read and no more than 3 mismatches.

### Identifying miRNA complementary sites on PHAS loci

The miRNA complementary sites on the original transcripts of PHAS loci were predicted with SeqTar. For conserved miRNAs, the targets that have at least one valid read, i.e., read started at the 9th to 11th positions of a miRNA binding site (as defined in [56]), or targets that have less than 4 mismatches were used for further analysis.

## Results and discussion

### RNA-seq profiles of leaves and petals of *R*.*rugosa*

Two petal and leaf samples of *R. rugosa* plants grown in Kunming University of Science and Technology, Yunnan, China were collected and put into liquid nitrogen immediately. Four RNA-seq profiles were generated using Illumina HiSeq 4000 sequencer. The qualities of these RNA-seq profiles are good (Additional file 2: Figure S1) after examining the scores of sequences with FASTQC. The RNA-seq profiles were assembled using Trinity (v2.0.6) [38]. We generated a self-assembled *R. rugosa* transcriptome with more than 37,000 segments. Then we use Trinity’s own program TrinityStats.pl to count the number and size of components and transcripts, N50, etc. As the result, we got 302,082 Trinity genes and 370,847 Trinity transcripts. The Contig N50 is 869. In order to derive annotation, we aligned this self-assembled transcriptome to NCBI nt/nr database (see “Methods” for details).

### Deregulated genes in leaves and petals of *R*.*rugosa*

**Fig. 1:**
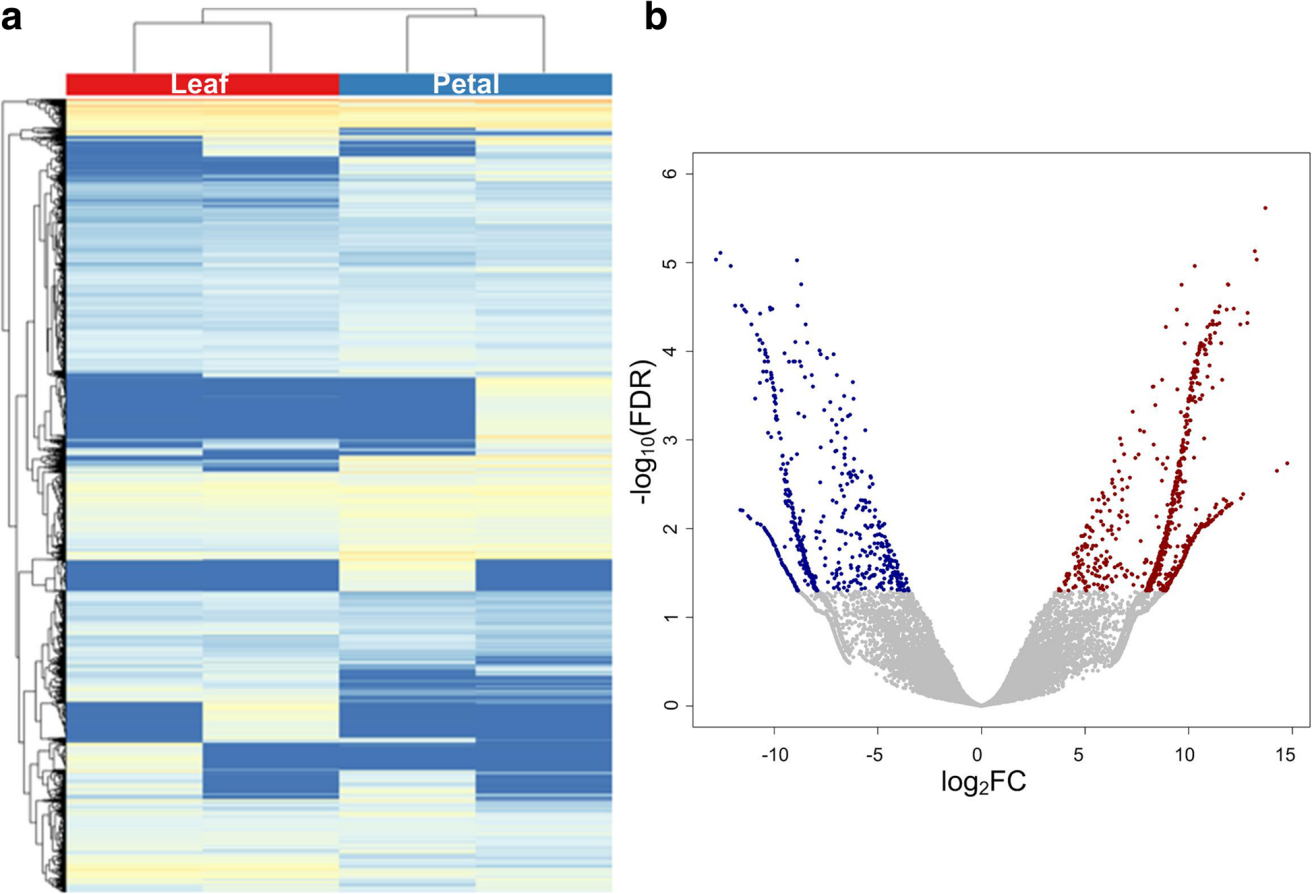
Expression patterns of genes in *R*.*rugosa* leaves and petals. **a** The bi-clustering of genes expression profiles in different tissues of *R. rugosa* RNA-Seq profiles. The values shown are the log2(FPTM+1) of the transcripts. **b** Deregulated genes when comparing their expression levels in petals to those in leaves. Up and downregulated genes in petals are shown in red and blue dots, respectively

**Fig. 2:**
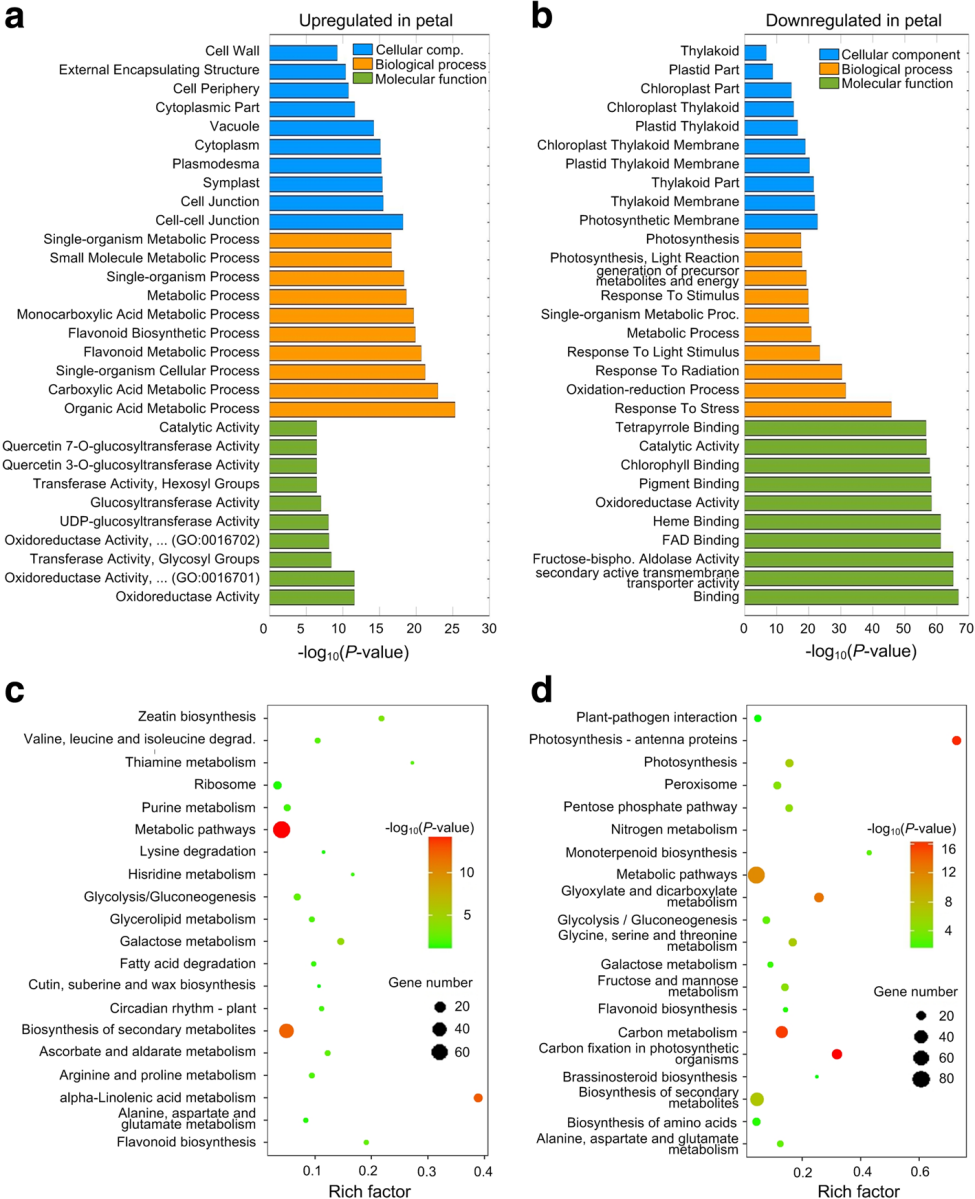
Enriched GO terms and KEGG pathways in deregulated genes of leaf and petal samples of *R*.*rugosa*. **a** GO terms of upregulated genes in petals. **b** GO terms of downregulated genes in petals. **c** Enriched KEGG pathways of upregulated genes in petals. **d** Enriched KEGG pathways of downregulated genes in petals. In Part (**c**) and (**d**), the Rich Factor is calculated by dividing the number of input genes with the KEGG pathway to the total number of genes with the same pathway

We calculated the normalized expression levels (Fragments Per Kilo basepairs per Million fragments mapped) of genes for four samples (Additional file 1: Table S3). Then, we performed Hierarchical Clustering based on the normalized frequencies of genes (Fig. 1a). We compared the abundances of leaves and petals with edgeR (Additional file 1: Table S4). We have identified 1811 deregulated genes. Among them, 1015 and 796 genes are upregulated and downregulated in petals, respectively (see Fig. 1b). These deregulated genes were used to analyze the enriched GO terms and KEGG pathways with KOBAS (version 3) [40]. The results show that the upregulated genes in petals are mainly involved in various metabolic processes and oxidoreductase (Fig. 2a), while the downregulated genes in petals, i.e., upregulated genes in leaves, are mainly involved in Response to Stresses, Oxidation-reduction Process, Response to Light Stimulus, and Binding (Fig. 2b). As shown in Fig. 2c and d, many upregualted and downregulated genes in petals are involved in Metabolic Pathway. Several pathways related photosynthesis, such as Photosynthesis - antenna proteins and Carbon fixation in photosynthetic organisms, are enriched in genes upregulated in leaves, i.e, downregulated in petals (Fig. 2d). These results are consistent with the types of the tissues, since the photosynthesis pathway is mainly performed in leaves.

### Small RNA sequencing profiles of different *R*.*rugosa* tissues

We collected the samples of roots, petals, pollens, stamens, leaves from *R. rugosa* that were grown in Kunming, Yunnan, China, without special treatments. Ten small RNA-seq (sRNA-seq) profiles, with two replicates for each of the five tissues, i.e., roots, petals, pollens, stamens, and leaves of *R. rugosa*, were generated using Illumina HiSeq 2000 sequencer (Additional file 1: Table S1). The qualities of these sRNA-seq profiles are good (Additional file 2: Figure S2) after examining the scores of sequences with FASTQC. Totally, we obtained 78,515,641 total reads represented by 25,456,782 unique small RNAs (Additional file 1: Table S2). The sRNA-seq profiles were aligned to the self-assembled transcriptome of *R. rugosa*, precursors of miRNAs in the miRBase (v21), other non-coding RNAs besides miRNAs, repeat elements and genome of *R. rugosa* with SOAP2 (see details in Methods) to obtain the distributions of sRNA reads (Additional file 2: Figure S3 and Additional file 1: Table S2).

### Identification of conserved miRNAs

**Fig. 3:**
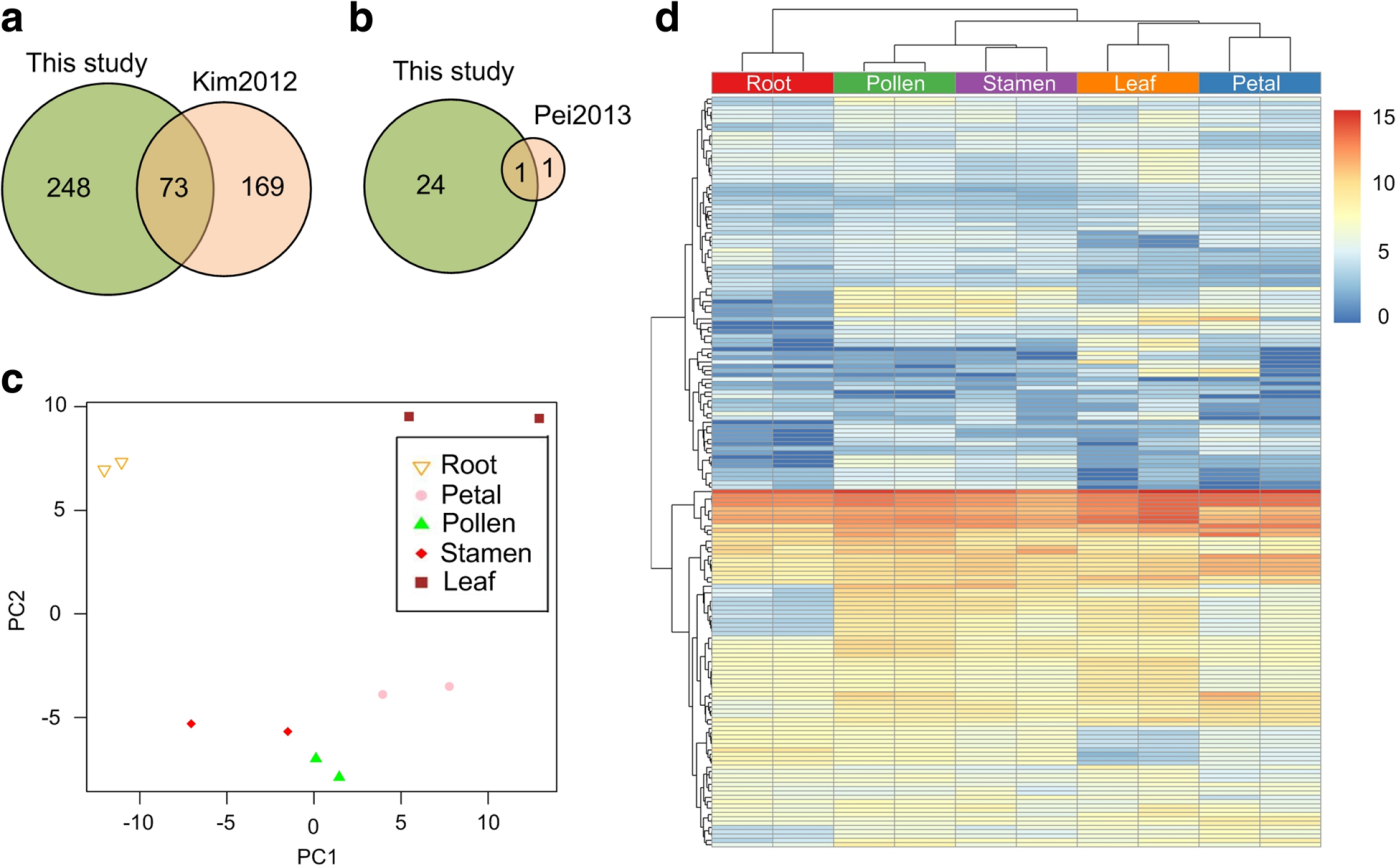
The conserved miRNAs in *R*.*rugosa* and their expression patterns in different tissues. **a** The number of conserved mature miRNAs identified in *R. rugosa*. **b** The number of pre-miRNAs identified in *R. rugosa*. **c** The PCA analysis of miRNA expression profiles in different tissues. **d** The bi-clustering of miRNA expression profiles in different tissues of *R. rugosa*. The values shown are the log2(RPTM+1) of the miRNAs

A total of 321 mature conserved miRNAs from other species were detected in our sRNA-seq profiles (Additional file 1: Table S5). A previous study [37] reported 242 conserved mature miRNAs, identified from flowers of three modern rose cultivars and *Rosarugosa*. Seventy three of the 242 miRNAs identified in this previous study were found in our libraries (Fig. 3a). In addition, we identified 248 conserved miRNAs that were not reported in their study (Fig. 3a). By aligning these mature miRNAs to the self-assembled *R. rugosa* transcriptome, we identified 25 precursors of conserved miRNAs (Additional file 1: Table S6 and Fig. 3b). Among them, only the precursor of miR482 had been reported previously [34]. When compared to two model species, *Arabidopsis thaliana* and rice, we found 18 members that belong to 11 miRNA families that are highly conserved (Additional file 1: Table S7).

### Expression patterns of conserved miRNAs in different tissues

Since we have constructed small RNA libraries from five different tissues of *R. rugosa*, we were able to analyze the different expression patterns of miRNAs in these tissues. Based on the frequencies of mature miRNAs after standardization, we performed PCA and Hierarchical Clustering. According to PCA and clustering results, it can be clearly seen that samples from the same tissue were clustered together, and samples from different tissues were distinguished. (Figure 3c and 3d). We also found that the expression levels of miRNAs are similar in the same tissues (Fig. 3d). These results indicate that miRNAs not only have important functions, but may also be involved in different physiological processes in different tissues.

To identify deregulated miRNAs in different tissues, we compared the expression levels of conserved miRNAs in samples from root, leaf, petal, stamen and pollen with edgeR [39] (Additional file 1: Tables S8 to S17). According to the results of differential expression analysis, we found 12 to 130 deregulated miRNAs between different tissues (Additional file 1: Table S18). For examples, MIR167j has higher expression levels in pollens, leaves, stamens than in the roots and petals; MIR319g has higher expression level in roots, petals, pollens and stamens than in leaves; and MIR156c has higher expression levels in pollens than roots, petals, and stamens.

### Discovery of novel miRNAs in *R*.*rugosa*

We identified 22 putative novel miRNAs in *R. rugosa* (Additional file 1: Table S19). Four of the 22 putative novel miRNAs are shown in Additional file 2: Figure S4. Both mature miRNA and miRNA* were detected in our sequencing libraries (Additional file 2: Figure S4a). The mature miRNAs demonstrated clear accumulated abundances than other regions of the pre-miRNAs in different sRNA sequencing libraries (Additional file 2: Figure S4b). These evidences suggest that these miRNAs are real miRNAs according to the criteria for annotating miRNAs [50].

### Identifying miRNA targets in *R*.*rugosa*

We producted a degradome profile with over 23 million reads (Additional file 1: Table S20) to identify miRNA targets. By running FASTQC, we found that most nucleotides of the degradome reads have sequencing scores of 35 or higher (Additional file 2: Figure S5). We used the SeqTar algorithm [56] to identify targets for conserved miRNAs from the obtained degradome profile of *R. rugosa* leaf. This analysis revealed more than 19,000 putative targets for conserved miRNA families (Additional file 1: Table S21). 592 of these targets have significant accumulation of reads at the identified miRNA complementary sites with at least 3 valid reads and *P*-value of valid reads smaller than 0.05 (Additional file 1: Table S21).

**Fig. 4:**
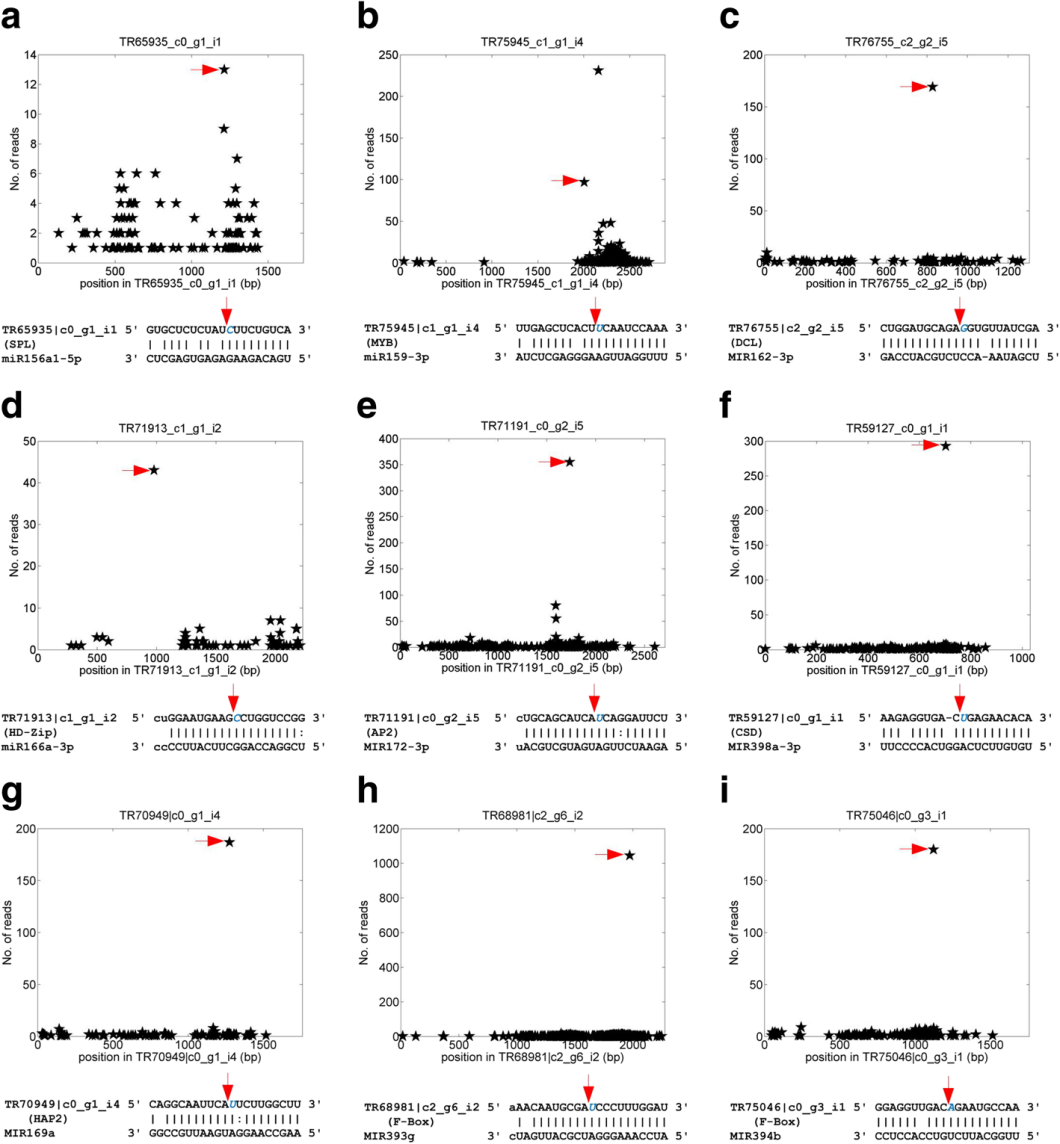
Some of the identified conserved targets of miRNAs in *R*.*rugosa*. The x-axis is the position on the transcript, and y-axis is the number of reads detected from a position. The arrows in the upper parts correspond to the positions pointed by the arrows of the same colors in the lower parts. **a** miR156a1-5p:TR65935 |c0_g1_i1, a SPL gene. **b** miR159-3p:TR75945 |c1_g1_i4, a MYB gene. **c** MIR162-3p:TR76755 |c2_g2_i5, a DCL gene. **d** miR166a-3p:TR71913 |c1_g1_i2, a HD-Zip gene. **e** MIR172-3p:TR71191 |c0_g2_i5, an AP2 gene. **f** MIR398a-3p:TR59127 |c0_g1_i1, a CSD gene. **g** MIR169a:TR70949 |c0_g1_i4, a HAP2 gene. **h** MIR393g:TR68981 |c2_g6_i2, a F-Box gene. **i** MIR394b:TR75046 |c0_g3_i1, a F-Box gene

We compared the number of conserved target genes for 26 highly conserved miRNA families to those in *Arabidopsis* and rice. Totally, we found 81 conserved targets for these 26 miRNA families (Additional file 1: Table S22). Nine of these targets were shown in Fig. 4 and 12 more were shown in Additional file 2: Figure S6. Among these targets identified in degradome analysis, only one AP2 gene targeted by miR172 (TR68272 |c1_g1_i3) was validated in a previous study [37].

### PHAS loci in *R*.*rugosa*

**Fig. 5:**
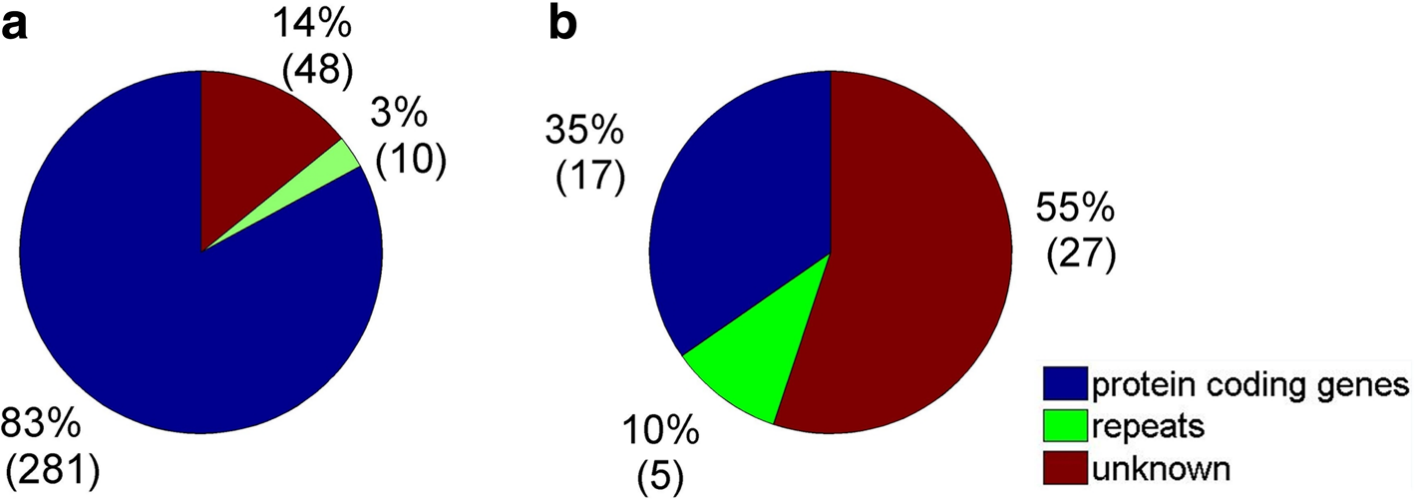
The types of molecules of the identified PHAS loci in *R*.*rugosa*. **a** The categories of 21 nt PHAS loci. **b** The categories of 24 nt PHAS loci

The sRNA profiles of 10 samples in this study were aligned to self-assembled *R. rugosa* transcriptome using SOAP2 [47]. Then, we used an in-house written program [57] to predict PHAS loci and phasiRNAs from the alignment results of SOAP2 based on methods described earlier (see Methods for details). We predicted three hundred and thirty nine 21 nt PHAS loci by using a phase score threshold of 5 and a multiple-test corrected *P*<0.05 (Additional file 1: Table S23). We aligned the obtained PHAS sequences to NCBI Nucleotide Collection (nr/nt) database and the TIGR Plant Repeat database to obtain putative annotation of the predicted PHAS loci (details are given in Additional file 1: Table S19). These PHAS loci were mainly derived from protein coding genes (Fig. 5a).

Similarly, by using a phase score threshold of 5 and a multiple-test corrected *P*<0.05, we predicted forty nine 24 nt PHAS (Additional file 1: Table S24). Most of 24 nt PHAS loci belong to unknown category in *R. rugosa* (Fig. 5b), which is different from the 21 nt PHAS loci. The second largest category of 24 nt PHAS loci is protein coding genes (Fig. 5b).

**Fig. 6:**
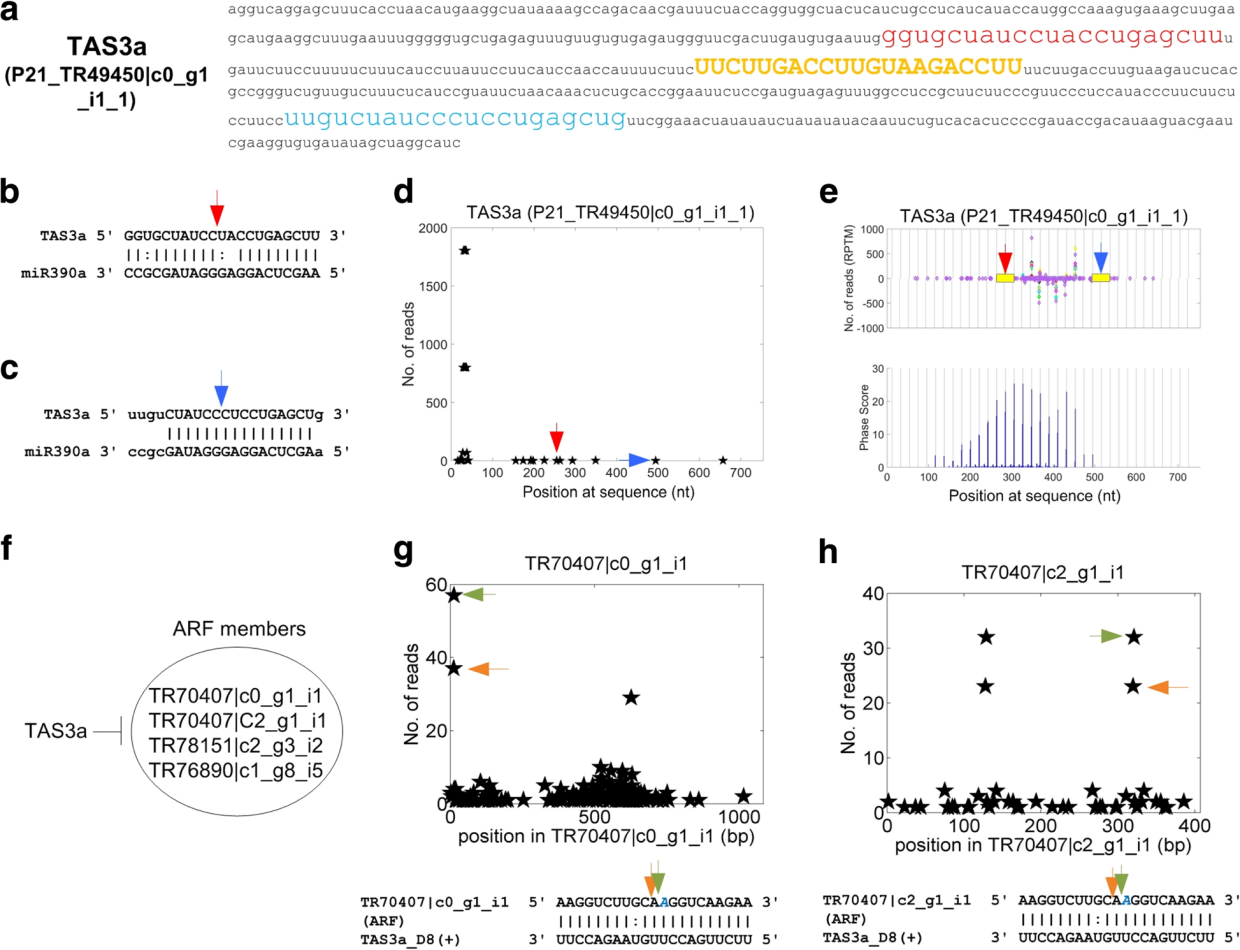
TAS3, tasiRNA and its targets in *R*.*rugosa*. **a** The sequence and genomic locus of TAS3a. The red and blue region are the 5’ and 3’ miR390 complementary sites. The yellow and black regions in upper cases are the tasiRNAs that target ARF genes. **b** The 5’ miR390 site on TAS3a. **c** The 3’ miR390 site on TAS3a. **d** The distribution of degradome reads on TAS3a transcript. The two positions pointed by red and blue arrows correspond to the 5’ and 3’ miR390 site shown in Part (**b**) and (**c**), respectively. **e** The distribution of small RNA reads in the small RNA profiles on TAS3a. **f** Four ARF genes that are targeted by TAS3 derived tasiRNAs. **g** The T-plot of and complementary sites of TAS3a_D8(+):TR70407 |c0_g1_i1. **h** The T-plot of and complementary sites of TAS3a_D8(+):TR70407 |c2_g1_i1. In Part (**g**) and (**h**), the x-axis is the position on the transcript, and y-axis is the number of reads detected from a position. The arrows in the upper parts correspond to the positions pointed by the arrows of the same colors in the lower parts

Taken together, the 339 and 49 PHAS loci encoding 2859 and 369 twenty one and twenty four nt phasiRNAs, respectively (Additional file 1: Tables S25 and S26, respectively). The putative targets of 21 and 24 nt phasiRNAs are provided in Additional file 1: Tables S27 and S28, respectively. One of the 21 nt PHAS loci is a TAS3 gene shown in Fig. 6a. It is targeted by miR390 at two loci (Fig. 6b and c) around a conserved tasiRNA, named as TAS3a_D8(+), (the yellow part in Fig. 6a), using the naming method described earlier [15]. From degradome analysis, it was found that a few reads corresponding to these two miR390 complementary sites were generated. This locus generates numerous siRNAs (Fig. 6d). The conserved tasiRNA targets at least four ARF genes (Fig. 6f). Two of these ARF targets are also validated by the degradome analysis (Fig. 6g and h).

Since miRNAs play key roles in the generation of phasiRNAs, we identified miRNA triggers for these PHAS loci on both strands by using the degradome sequencing profile and the SeqTar algorithm. The putative miRNA triggers of PHAS loci are also listed in Additional file 1: Table S23.

**Fig. 7:**
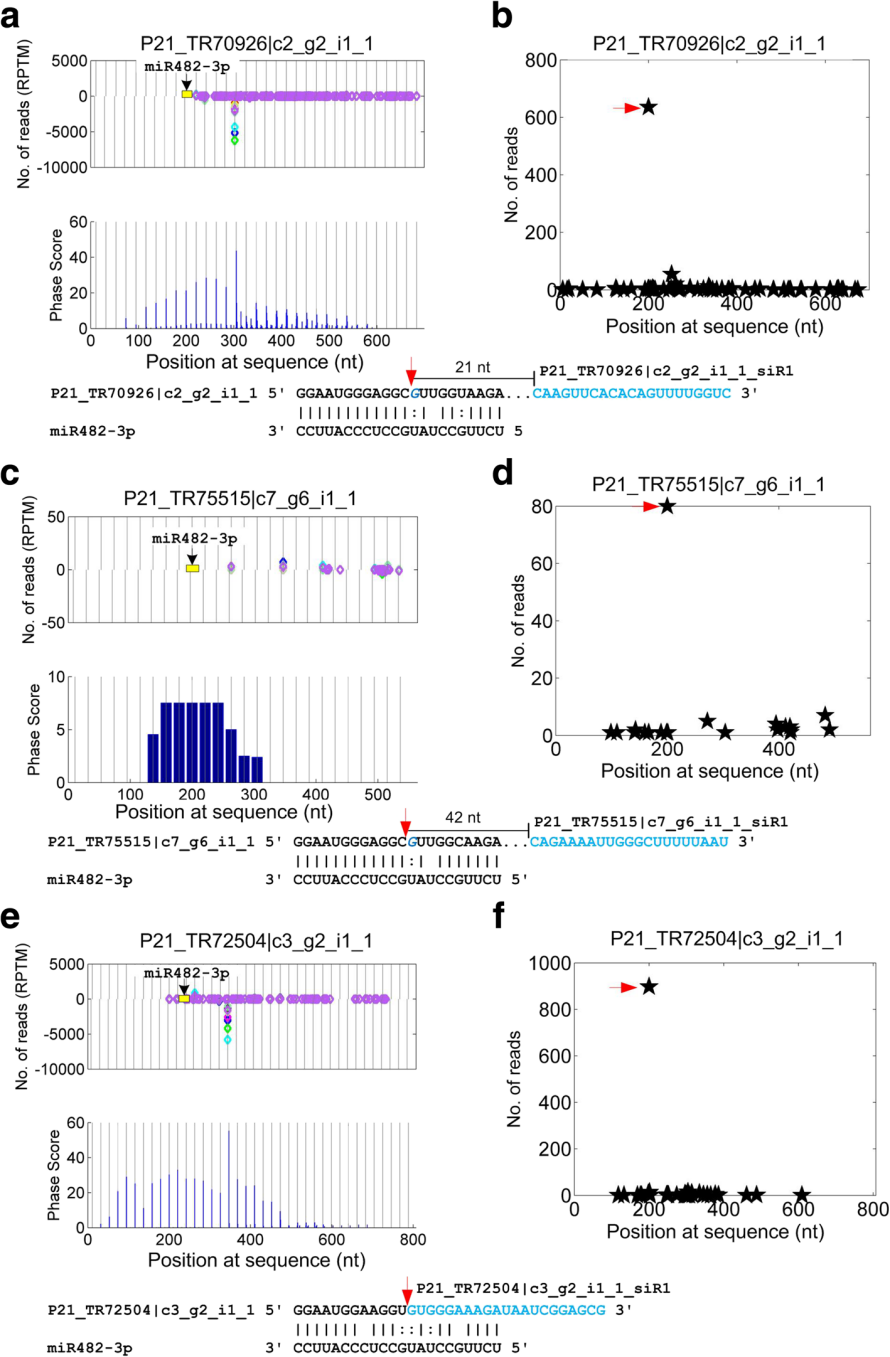
Three PHAS loci from NB-LRR genes that are triggered by miR482. **a** The distribution of 21 nt siRNAs and Phase Scores on P21_TR70926 |c2_g2_i1_1. **b** The T-plot of and complementary sites of miR482a-3p:P21_TR70926 |c2_g2_i1_1. **c** The distribution of 21 nt siRNAs and Phase Scores on P21_TR75515 |c7_g6_i1_1. **d** The T-plot of and complementary sites of miR482a-3p:P21_TR75515 |c7_g6_i1_1. **e** The distribution of 21 nt siRNAs and Phase Scores on P21_TR72504 |c3_g2_i1_1. **f** The T-plot of and complementary sites of miR482a-3p:P21_TR72504 |c3_g2_i1_1. In Part (**a**), (**c**) and (**e**), the vertical gray lines with distances of 21 nt are the phased positions from the position with the highest phase scores of the PHAS loci. The yellow boxes in the read distribution panel represent the miR482a-3p complementary sites. Sites pointed by miRNAs from above and under zero read line means miRNAs complement to the plus and minus strand of the predicted PHAS loci, respectively. The predicted miR482a-3p complementary sites are shown below the phase score panel. The blue region after the complementary sites are one of the phasiRNAs generated from the PHAS loci

It is worth mentioning that miR482 potentially triggers the generations of phasiRNAs from more than 20 NB-LRR genes (Additional file 1: Table S23). Two of them are shown in Fig. 7. From Fig. 7a, c and e, it is shown that miR482-3p triggers the generations of siRNAs from the centers of the complementary sites. Meanwhile, as indicated in Fig. 7b, d and f, miR482-3p could induce strong cleavages on these PHAS transcripts.

## Conclusion

*R. rugosa* is an important crop plant for economical and floricultural reasons. However, there are only few studies that reported sRNA profiles in this important plant species. We systematically sequenced and analyzed 10 small RNA libraries from five different tissues of *R. rugosa* from which 321 conserved mature miRNAs were detected. We also identified 25 pre-miRNAs, 339 PHAS loci encoding 21 nt phasiRNAs, and 49 PHAS loci encoding 24 nt phasiRNAs. We also identified 22 novel miRNAs. By analyzing a degradome library, more than 500 reliable targets of conserved miRNAs or tasiRNAs were identified. Furthermore, our results suggest that approximately 20 putative NB-LRR genes are targeted by miR482-3p to generate phasiRNAs in *R. rugosa*. The RNA-seq profiles of leaves and petals suggest that thousands of genes have shown significantly different expression levels in these two tissues of *R. rugosa*, and these genes are enriched in GO terms and KEGG pathways related to metabolic processes and photosynthesis. These results significantly improved our knowledge of sRNA guided gene regulations in *R. rugosa*.

## Additional files

Additional file 1 This is an MS Excel file. This file includes 28 supplementary tables. (XLSX 43750 kb)

Additional file 2 This is a pdf file. This file includes 6 supplementary figures. (DOCX 1106 kb)

## Abbreviations

AGO: Argonaute
AP2: Adipocyte protein 2
ARF: Auxin response factor
CC: Correlation coefficient
cDNA: Complementary DNA
CSD: Copper-zinc superoxide dismutase
DCL: Dicer like
DCL1: Dicer like-1
DCL4: Dicer like-4
DCL5: Dicer like-5
dsRNAs: double strand RNAs
FDR: False discovery rate
FPKM: Fragments per kilobase million sequencing tags
GO: Gene ontology
HAP2: HAPLESS2
HD-Zip: Homeodomain leucine zipper family of transcription factors
KEGG: Kyoto encyclopedia of genes and genomes
logFC: log2FoldChange
miRNAs: microRNAs
mRNAs: message RNAs
MYB: Myeloblastosis family of transcription factors
NB-LRR: Nucleoside-binding site leucine rich repeat genes
ncRNAs: non-coding RNAs
PCA: Principle component analysis
phasiRNAs: phased small interfering RNAs
PPR: Pentatricopeptide repeats
pre-miRNAs: precursor microRNAs
RDR6: RNA-dependent RNApolymerase6
RISC: RNA-induced silencing complex
RNA-seq: RNA sequencing
RPTM: Reads per ten million sequencing tags
SeqTar: SEQuencing-based sRNA TARget prediction siRNAs: Small interfering RNAs
siRNAs: small interfering RNAs
SOAP: Short oligonuclotide analysis program
SPL: SPOROCYTELESS
sRNA: small RNA
sRNA-seq: small RNA-seq
tasiRNAs: trans-acting small interfering RNAs
