# Supplementary material for: Analysis of microRNAs, phased small interfering RNAs and their potential targets in Rosarugosa Thunb
Source: BMC Genomics. 2019 Apr 18;19(Suppl 9):983. doi: 10.1186/s12864-018-5325-2 (PMC7394236; doi:10.1186/s12864-018-5325-2)
Supplement: Supplementary file 2 — This is a pdf file. This file includes 6 supplementary figures. (DOCX 1106 kb) [file 12864_2018_5325_MOESM2_ESM.docx]

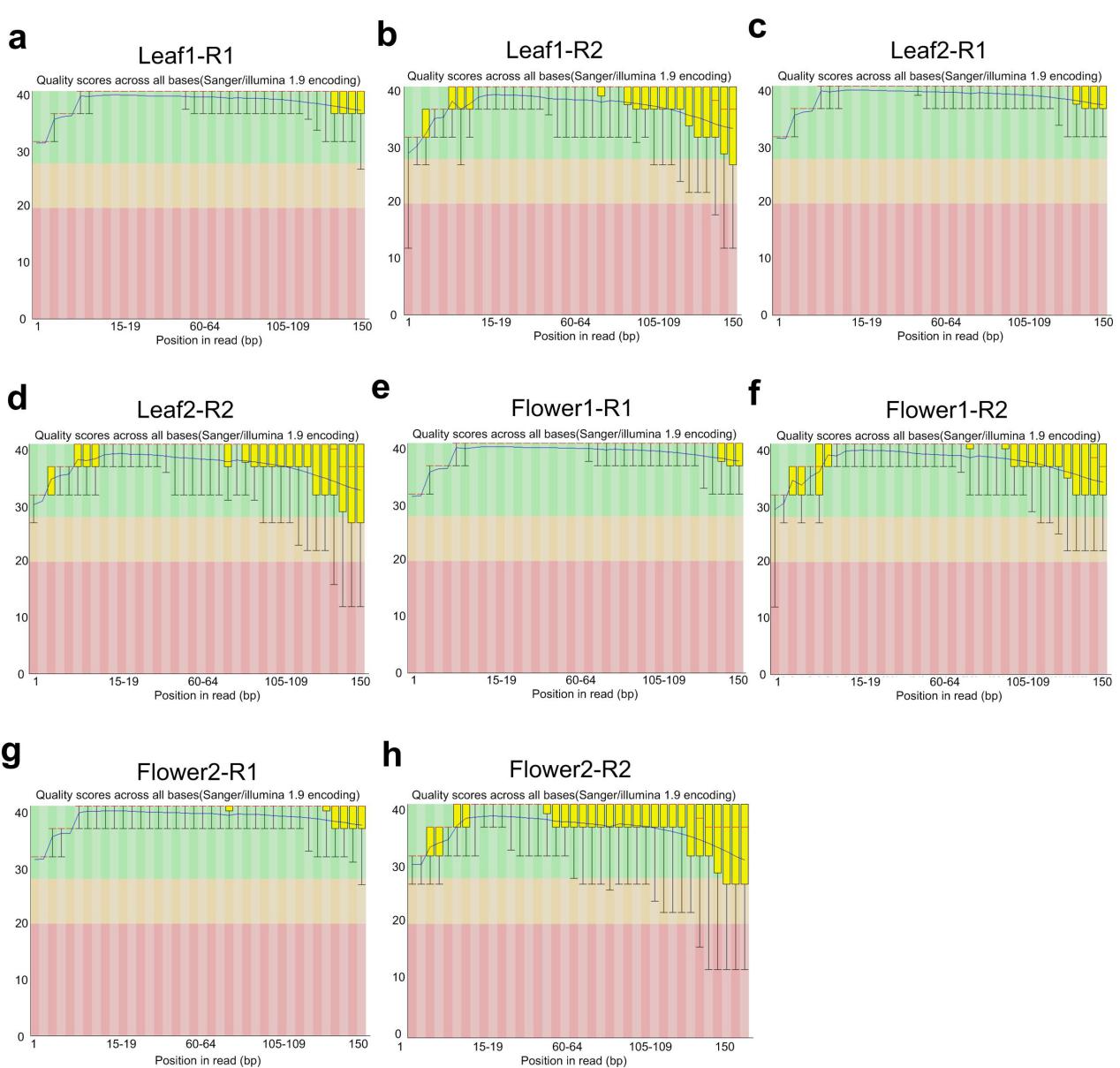


**Figure S1.** Per base sequence quality of 4 RNA-Seq sequencing profiles.


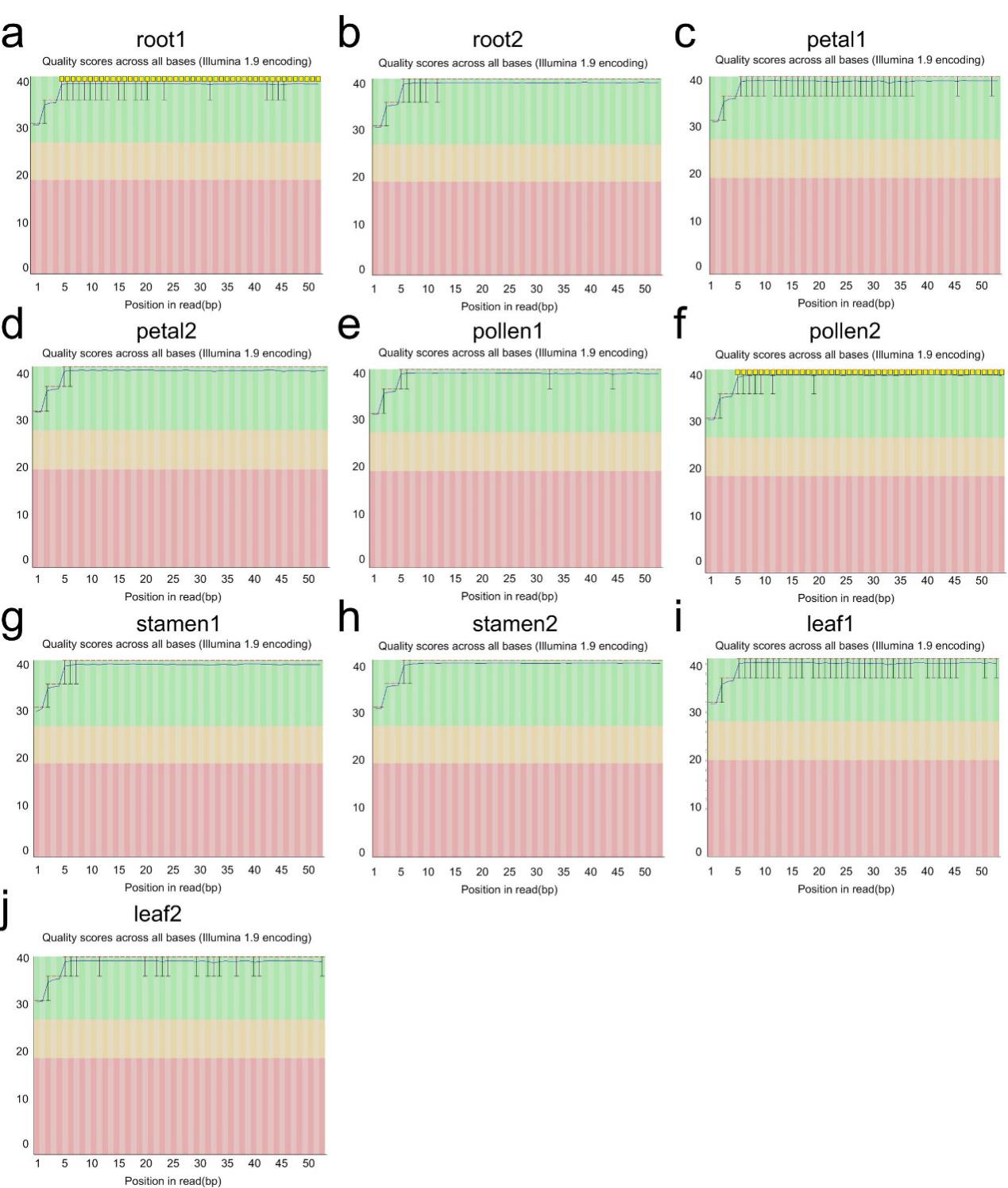


**Figure S2.** Per base sequence quality of 10 small RNA sequencing profiles.


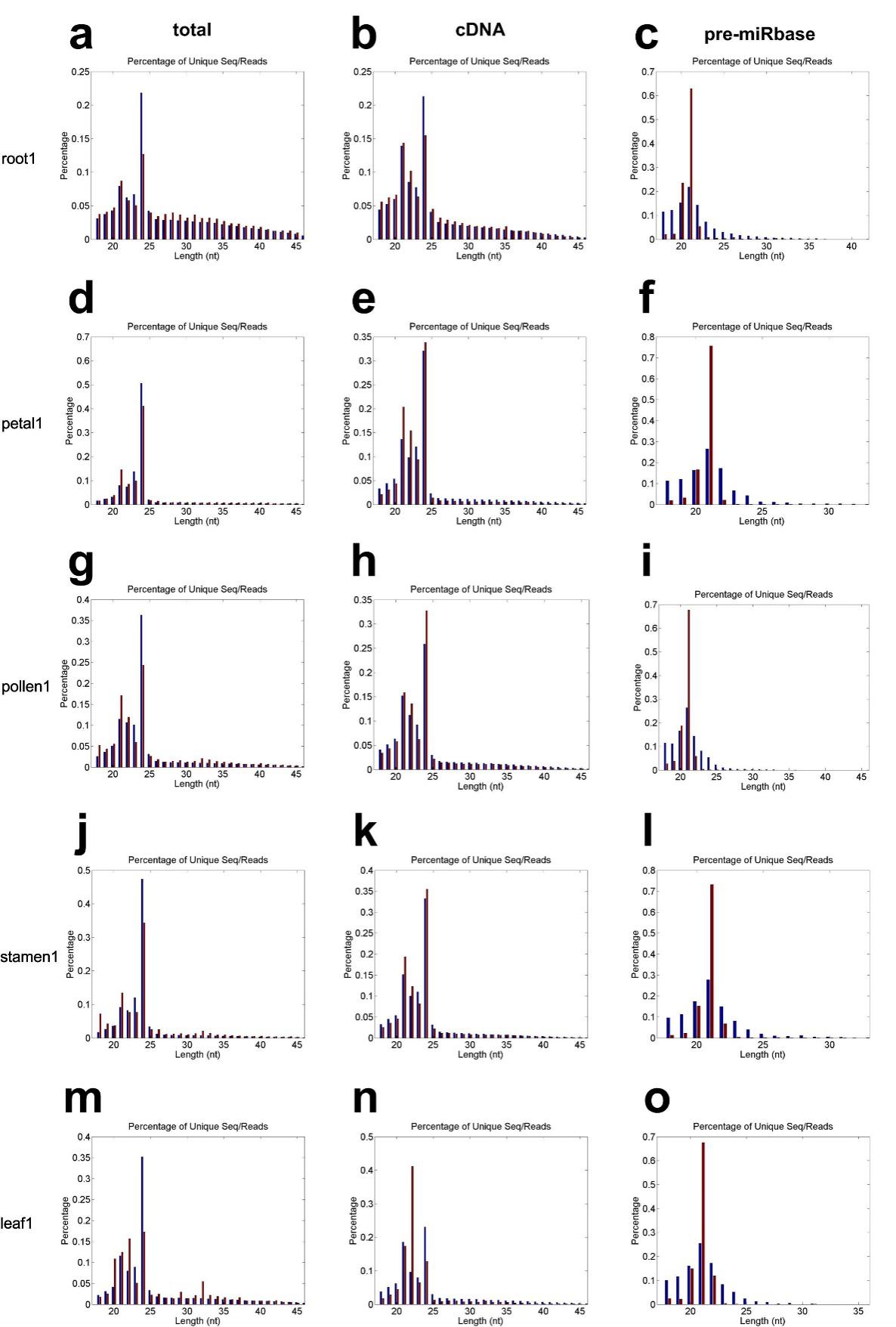


**Figure S3.** The length distributions of reads and unique sequences in 5 sRNA-seq libraries. (a) - (c) are the first root sample. (d) - (f) are the first petal sample. (g) - (i) are the first pollen sample. (j) - (l) are the first stamen sample. (m) - (o) are the first leaf sample. The vertical axis show the percentages of reads (red bars) and unique sequences (blue bars). The first column lists the distributions for reads and unique sequences in the whole libraries. The second column lists the distributions for reads mapped to genome. The third column lists the distributions for reads mapped to pre-miRBase.


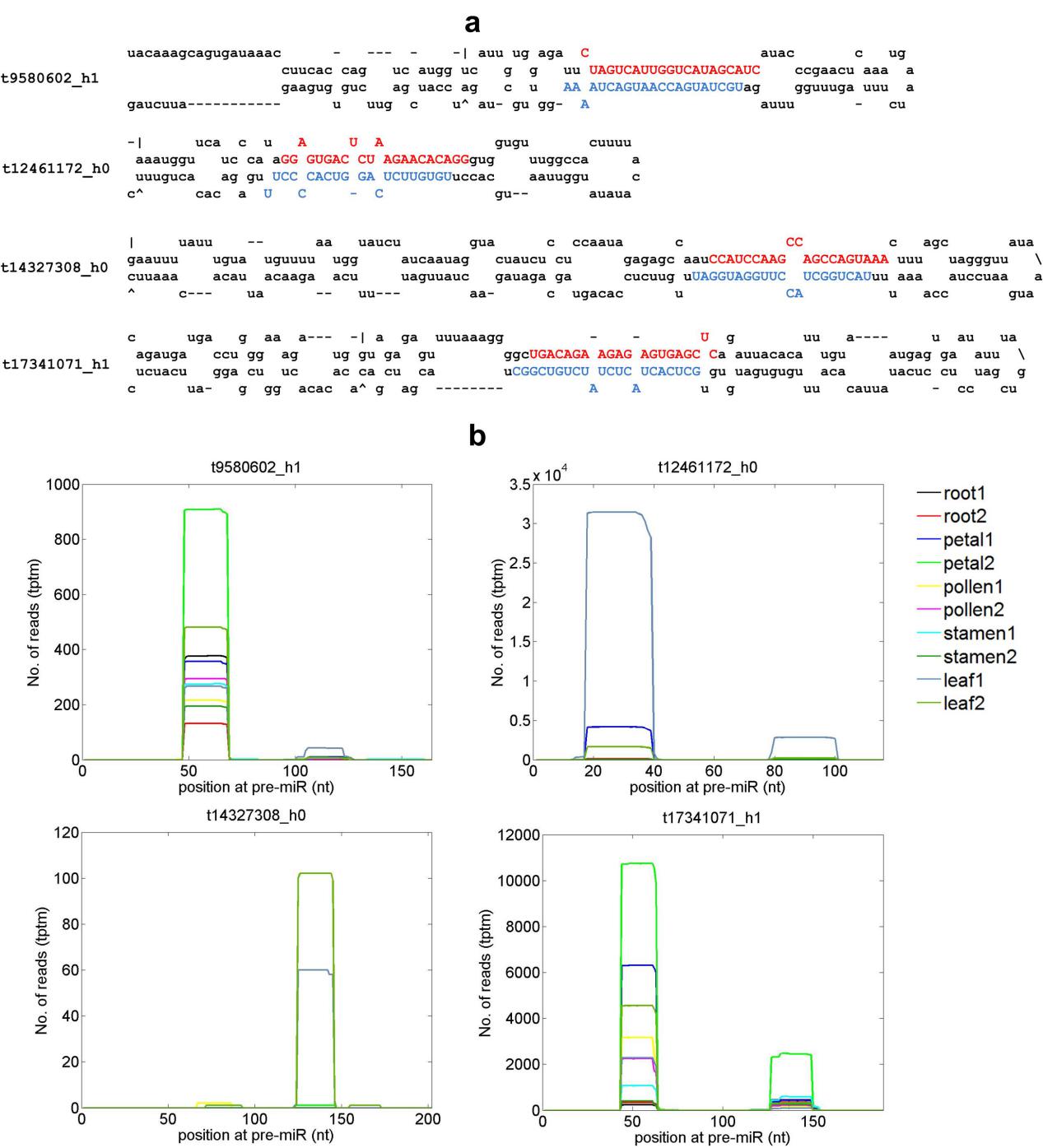


**Figure S4.** Four of the identified novel miRNAs in rose. (a) The secondary structures of three novel miRNA precursors. (b) The distribution of reads on the four novel pre-miRNAs, The number of reads are normalized to Tags Per Ten Million (RPTM) sequencing.
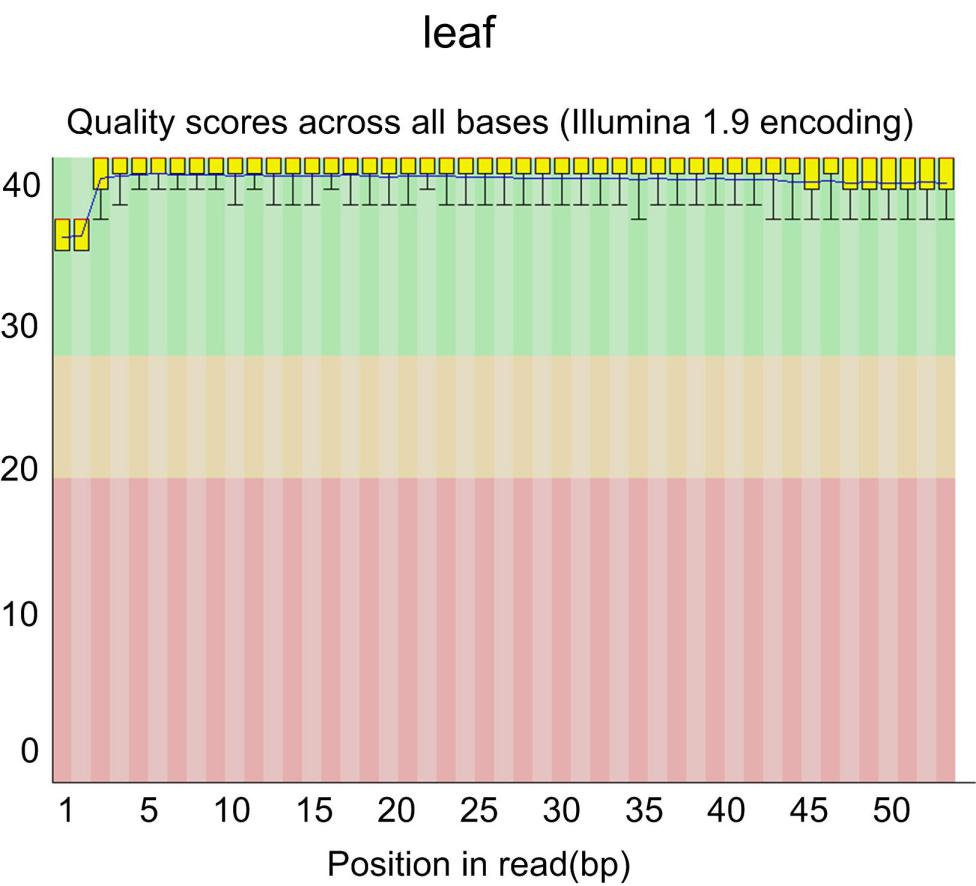


**Figure S5.** Per base sequence quality of degradome sequencing profile.


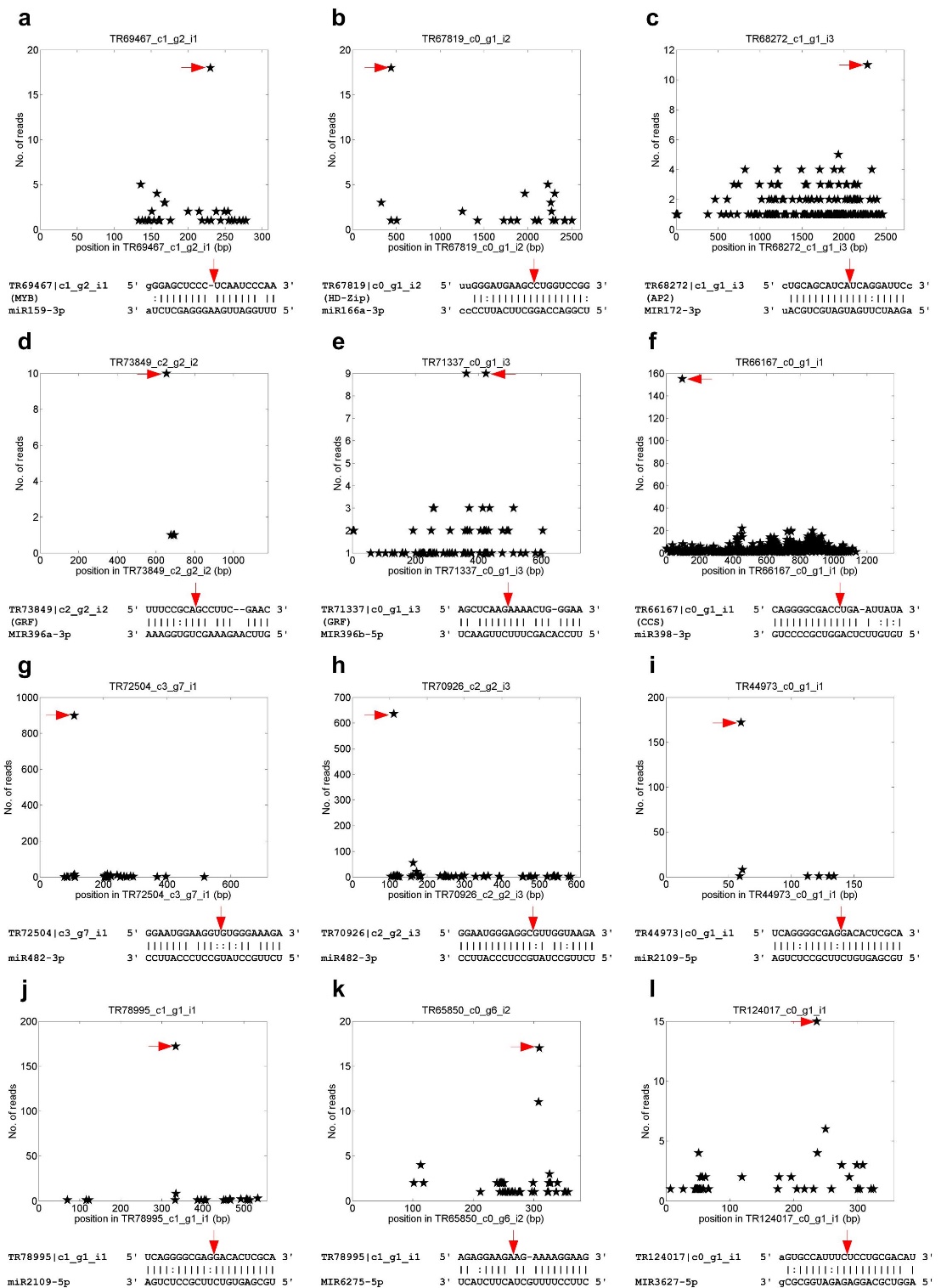


**Figure S6.** Some of the identified conserved miRNA targets. The x-axis is the position on the transcript, and y-axis is the number of reads detected from a position. The arrows in the upper parts correspond to the positions pointed by the arrows of the same colors in the lower parts. (a) miR159-3p: TR69467|c1_g2_i1, a MYB transcription factors gene. (b) miR166a-3p: TR67819|c0_g1_i2, a HD-Zip transcription factors gene. (c) MIR172-3p: TR68272|c1_g1_i3, an AP2 transcription factors gene. (d) MIR396a-3p: TR73849|c2_g2_i2, a GRF gene. (e) miR396b-5p: TR71337|c0_g1_i3, a GRF gene. (f) miR398-3p: TR66167|c0_g1_i1, a CCS gene. (g) miR482-3p: TR72504|c3_g7_i1. (h) miR482-3p: TR70926|c2_g2_i3. (i) miR2109-5p: TR44973|c0_g1_i1. (j) miR2109-5p: TR78995|c1_g1_i1. (k) MIR6275-5p: TR78995|c1_g1_i1. (l) MIR3627-5p: TR124017|c0_g1_i1.
